# Nanodroplet-Vaporization-Assisted Sonoporation for Highly Effective Delivery of Photothermal Treatment

**DOI:** 10.1038/srep24753

**Published:** 2016-04-20

**Authors:** Wei-Wen Liu, Shu-Wei Liu, Yu-Ren Liou, Yu-Hsun Wu, Ya-Chuen Yang, Churng-Ren Chris Wang, Pai-Chi Li

**Affiliations:** 1 National Taiwan University, Graduate Institute of Biomedical Electronics and Bioinformatics, Taipei 106, Taiwan; 2 National Chung-Cheng University, Department of Chemistry and Biochemistry, Chia-Yi 621, Taiwan; 3 National Taiwan University, Department of Electrical Engineering, Taipei 106, Taiwan

## Abstract

Sonoporation refers to the use of ultrasound and acoustic cavitation to temporarily enhance the permeability of cellular membranes so as to enhance the delivery efficiency of therapeutic agents into cells. Microbubble-based ultrasound contrast agents are often used to facilitate these cavitation effects. This study used nanodroplets to significantly enhance the effectiveness of sonoporation relative to using conventional microbubbles. Significant enhancements were demonstrated both *in vitro* and *in vivo* by using gold nanorods encapsulated in nanodroplets for implementing plasmonic photothermal therapy. Combined excitation by ultrasound and laser radiation is used to trigger the gold nanodroplets to induce a liquid-to-gas phase change, which induces cavitation effects that are three-to-fivefold stronger than when using conventional microbubbles. Enhanced cavitation also leads to significant enhancement of the sonoporation effects. Our *in vivo* results show that nanodroplet-vaporization-assisted sonoporation can increase the treatment temperature by more than 10 °C above that achieved by microbubble-based sonoporation.

Sonoporation is the process that involves microbubbles (MBs) interacting with ultrasound to facilitate drug or gene uptake into the cells. Sonoporation can temporarily increase the permeability of a tumor cell membrane or open the tight junctions of endothelial walls to deliver drugs, genes, or other therapeutic agents into the tumor lesion^1^, and it is closely associated with stable cavitation and inertial cavitation^2^. MBs undergo stable oscillation—also named stable cavitation—when they are exposed to a low acoustic pressure^2^. Such stable oscillations produce small-scale fluid swirls surrounding the MBs, termed acoustic microstreaming^3^,^4^. Such MBs in close contact with cells can disrupt the cellular tight junctions by the shear stress resulting from the MBs contracting and expanding^5^,^6^. When MBs collapse violently under exposure to a high acoustic pressure, known as inertial cavitation, membrane or vascular pores are created by the shock-wave-induced shear stress and microjetting^7^,^8^,^9^. This transiently increase in intracellular permeability derived from sonoporation thus enhances the delivery of therapeutic reagents^10^.

The enhancement efficacy of ultrasound-directed drug delivery has been greatly influenced by advances in contrast agents^11^. The most popular type of contrast agent is MBs, which have been developed over many years and are usually applied to facilitate sonoporation in drug delivery and to enhance ultrasound imaging contrast. For example, photoacoustics-based theranosis utilizes gold nanorods (AuNRs)-encapsulated microbubbles (AuMBs) as a photoacoustic dual-modality contrast agent, and we have applied this method in a previous study^12^. The use of *in vivo* sonoporation for facilitating the delivery of drugs or genes has been widely studied in recent years, with most studies using MBs as the carrier for drugs or genes, which combined with the inertial cavitation effect increases the vascular and cellular permeability for the delivery of drugs or genes across the vascular wall or cellular membrane^2^,^13^,^14^. Sonoporation can also be used to effectively deliver drugs or small molecules across the blood–brain barrier in applications such as therapy for central nervous system diseases^1^.

Despite the widespread use of MBs for sonoporation, some problems may occur during drug delivery such as drug loss due to gas diffusion or unstable MB collapse caused by shear forces generated in the bloodstream^15^,^16^. Moreover, MBs are on average 1–3 μm in diameter, making them too big to extravasate into the tumor tissue from the endothelial gaps of the leaky tumor vessel^17^, which decreases the tumor therapeutic efficiency due to impairment of the enhancement of permeability and retention (EPR) effects.

With the aim of overcoming these limitations of MBs, nanodroplets (NDs) have been developed as a new drug carrier due to their higher stability and smaller size^18^. NDs can be synthesized with an albumin, lipid, or polymer shell, and are usually filled with a liquid perfluorocarbon. The low boiling point of perfluorocarbons means that NDs become superheated during circulation under body temperature, which makes them easy to vaporize^19^. The droplet-to-bubble phase transition property of NDs has been utilized in several applications as described below, and such phase-shift vaporization can be triggered either acoustically or optically^20^,^21^. NDs have been exploited for renal occlusion therapy, drug delivery enhancement, and assisting opening of the blood–brain barrier^16^,^22^,^23^,^24^. NDs have also been combined with high-intensity focused ultrasound to implement and enhance sonothrombolysis and the thermal ablation of tumors^25^,^26^,^27^,^28^,^29^,^30^,^31^. NDs can also be used to enhance sonoporation by permeabilizing the vascular wall during excitation by continuous-wave ultrasound^31^. One recent report also indicates that NDs can serve as a DNA carrier for cellular gene transfection^33^, and the successful release of microRNA and small interfering RNA from NDs to cancer cells has also been reported^34^,^35^. In addition to therapeutic applications, it has been demonstrated that gold nanoparticles (AuNPs)-encapsulated NDs can produce photoacoustic signals that are 100 times higher than the signals produced by gold nanoparticles alone^36^.

By transforming photon energy into heat energy, photothermal therapy can cause irreversible damage in tumor cells by elevating the temperature of the tissue to over 42 °C. Effective photothermal therapy can be achieved by utilizing AuNPs as a therapeutic agent, which is also called plasmonic photothermal therapy (PPTT)^37^. By converting electromagnetic energy into heat energy via the local surface plasma resonance induced by light, the electrons of AuNPs are excited so as to rapidly elevate the temperature of a tumor mass^37^. Injecting targeted AuNPs into a tumor site following laser radiation can cause tumor necrosis^38^. However, the therapeutic efficiency is limited by it being difficult to accumulate large numbers of AuNPs in tumor tissue. Although MBs have been used as a carrier for delivering AuNPs to targeted tumor tissues when conducting PPTT, both the delivery efficacy and the accumulation of AuNPs have remained inadequate in most cases. Specifically, despite various studies having demonstrated the advantages of NDs over MBs, encapsulating AuNPs into NDs for the effective delivery of AuNPs followed by PPTT has not been reported previously.

In our previous study, we successfully applied dual-modality targeting MBs comprising encapsulated AuNRs with an albumin shell and gas-filled core for enhancing the efficiency of AuNRs delivery through active targeting and sonoporation^15^. Although that work demonstrated the ability to enhance the therapeutic efficiency, due to the limitations of gas-filled MBs we proposed replacing them with NDs in order to increase the vehicle stability and exert stronger cavitation effects to achieve enhanced extravasation for the delivery of AuNRs. The present study developed NDs composed of a low-boiling-point dodecafluorocarbon (DDFC) liquid core and a human serum albumin (HSA) protein shell. The efficiency of liquid-to-gas vaporization-assisted sonoporation when using NDs as AuNPs carriers was assessed. We also evaluated the therapeutic efficiency of cavitation-based, gold nanodroplets (AuNDs)-assisted PPTT both *in vitro* and *in vivo*. Finally, the synergy among various mechanisms by which sonoporation facilitates the AuNDs-assisted PPTT was investigated.

## Results

### Characterization of AuNDs

**Figure 1:**
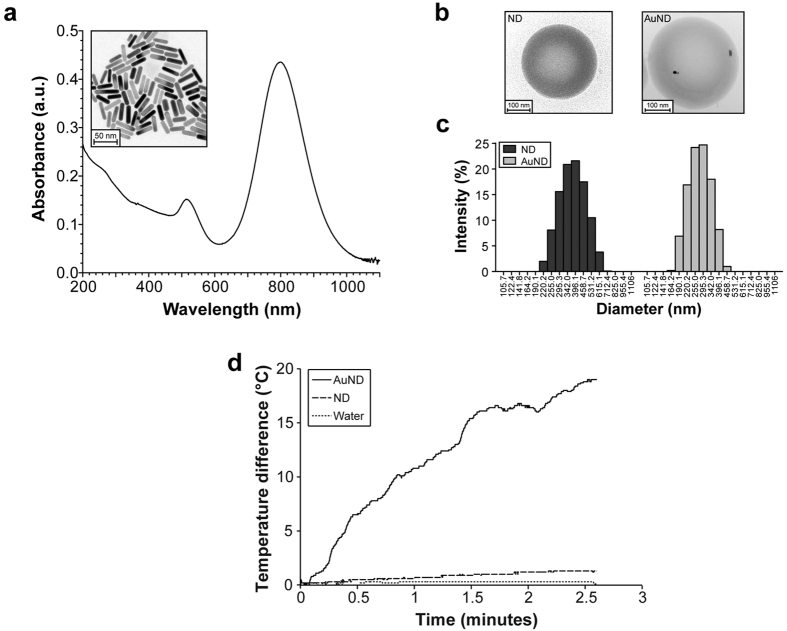
Characterization of AuNDs. (**a**) The optical absorption peaked at 808 nm. Inset TEM image accurately shows the aspect ratio of rod-shaped AuNRs. (**b**) cTEM images of NDs and AuNDs. (**c**) Size distributions of NDs and AuNDs. (**d**) Temporal temperature profiles for water, NDs, and AuNDs during exposure to continuous-wave laser radiation.

Nanosized AuNDs with a liquid dodecafluorocarbon core and an HSA shell were synthesized using an emulsion method. The photoresponsive agent encapsulated in the NDs was AuNRs with a plasmonic surface so as to maximize the collective oscillations of electrons. To check whether the optical properties of the AuNRs were specifically tuned, the size distribution of AuNRs was determining under transmission electron microscopy (TEM) and the optical density (OD) of the AuNRs was investigated using spectrophotometry. TEM indicated that the AuNRs had an average aspect ratio of four—th’e rods were around 12 nm in diameter and had a length of 48 nm (Fig. 1a). Absorption wavelength measurements indicated that the longitudinal plasmon band of the AuNRs peaked at 813 nm. Cryogenic TEM (cTEM) was used to investigate the spherical structure of the AuNDs to ensure that the AuNRs had been successful encapsulated within the synthesized AuNDs. NDs and AuNDs were sectioned and then examined under TEM, which clearly showed that AuNRs were successfully encapsulated and randomly distributed in AuNDs (Fig. 1b). The size distributions of NDs and AuNDs were accurately measured utilizing dynamic light scattering, which revealed that the sizes of more than 70% of the NDs and AuNDs were distributed around 250–450 nm and 220–340 nm, respectively (Fig. 1c). Nanosized AuNDs produced according to the emulsion method followed by differential centrifugation could be isolated. To confirm that the AuNDs were exhibiting optical absorbance at 808 nm (i.e., in the near-infrared region), the temperature of AuNDs exposed to laser radiation was monitored. In comparison with controls comprising water and ND without AuNRs encapsulation, the temperature of AuNRs-encapsulated AuNDs increased rapidly within 2.5 minutes (Fig. 1d). These results indicate that the temperature of AuNDs was effectively elevated through the conversion of photon energy into heat by exposure to 808-nm near-infrared radiation.

### Optically triggered vaporization of AuNDs

**Figure 2:**
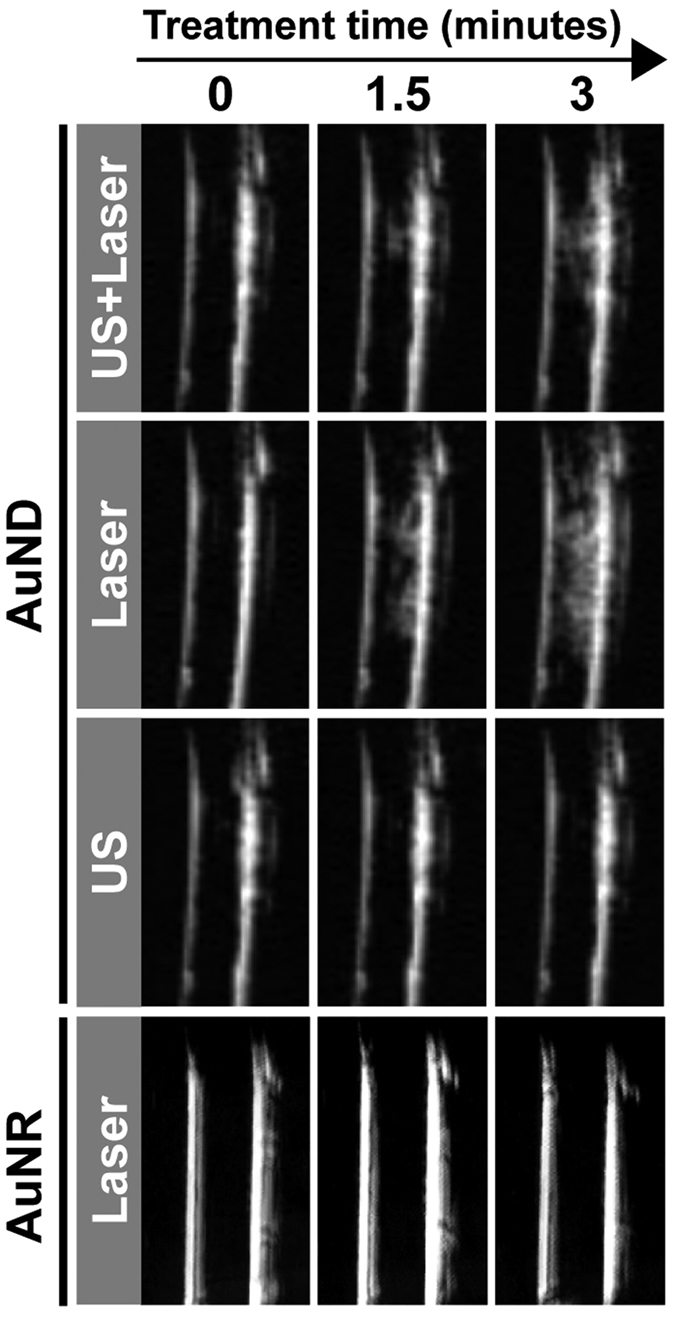
Vaporization of AuNDs. B-mode images showing the temporal profile of the vaporization of AuNDs during exposure to ultrasound only, laser radiation only, ultrasound plus laser radiation, or AuNRs exposed to laser radiation only at 0 minutes, 1.5 minutes, and 3 minutes (left, middle, and right panels). The two white near-vertical linear areas in each image correspond to the walls of the tubes.

The vaporization of DDFC AuNDs by optical stimulation generates strong acoustic signals which can be used for ultrasound contrast enhancement^36^. Thus, we used B-mode imaging to monitor the vaporization of AuNDs (Fig. 2). The two white near-vertical linear areas in each B-mode image correspond to the walls of the tubes, and the presence of AuNDs vaporization is indicated by white areas between the tube walls. The efficacy of AuNDs vaporization was highest for the laser-exposed tube, while the vaporization was barely present in the ultrasound-exposed one. It was also found that even when the vaporization was triggered by the laser radiation and ultrasound concurrently, it was not as obvious as when it was triggered only by the laser radiation. This indicates that the vaporization was triggered mainly by the laser radiation rather than by the ultrasound.

### Acoustic cavitation effect and AuNRs release

**Figure 3:**
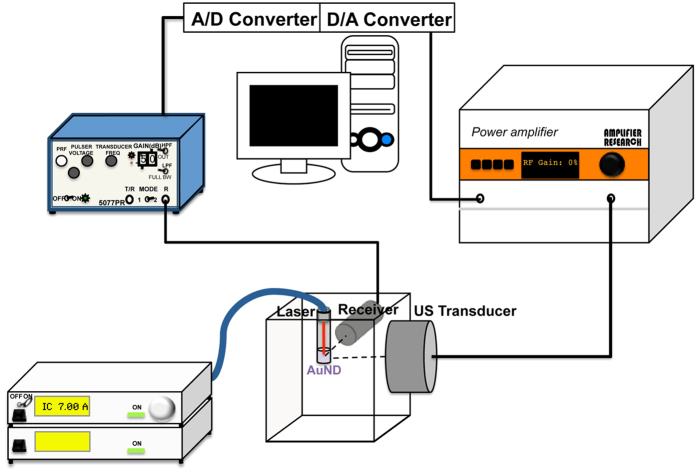
System setup for ICD measurement. The diagram shows the system setup for ICD value measurements *in vitro*. A/D converter, analog-to-digital converter; D/A converter, digital-to-analog converter.

**Figure 4:**
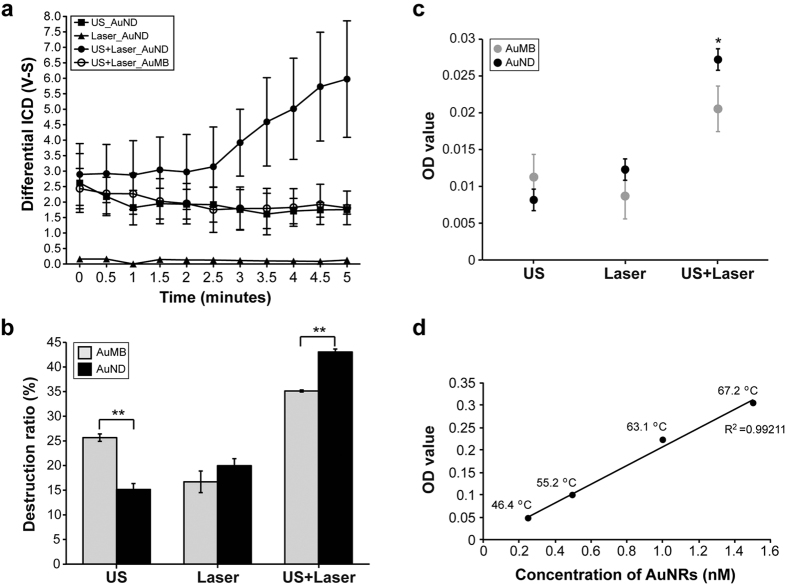
Enhanced cavitation effect and efficiency of AuNRs release. (**a**) Differential ICD–time curve showing the change in the cavitation effect induced by the indicated contrast-agent-assisted energy application methods. Upon exposure to ultrasound and laser radiation simultaneously for 5 minutes, the dICD value was enhanced by 3.3 fold for AuNDs compared to AuMBs with the same treatment, and it was enhanced by 3.4 fold compared to AuNDs exposed to ultrasound only. The time zero (t = 0) labeled at the X axis is the starting time to transmit the ultrasound signal from the 1 MHz transmitting transducer, and the amount of cavitation signals are collected by the 10 MHz receiving transducer with a 13-seconds delayed. (**b,c**) The destruction ratio and the rate of AuNRs release were measured after AuNDs or AuMBs were exposed to the indicated energy modalities. The initial OD values of the same number of the intact AuNDs or AuMBs before exposed to the indicated energy modalities were 0.084 ± 0.006 or 0.068 ± 0.009 (mean ± SD), respectively. Statistically significant differences between two independent groups were determined using the statistical methods described in the Methods. Data are mean and SD values. *p < 0.05. **(d)** The plot showing the OD values function to the different concentrations of free AuNRs. Temperatures labeled near the black circles indicate the final temperatures after indicated concentration of free AuNRs was exposed to laser radiation for 5 minutes. The initial temperature before the treatment is 18.2 °C. The R-squared is 0.99211 and the equation for the best fit regression line is y = 0.2089x-0.002.

Inertial cavitation is a physical phenomenon in which vapor bubbles collapse violently at sufficient acoustic pressures. To confirm that inertial cavitation was generated when optically vaporized AuNDs were exposed to ultrasound, the cavitation signals were measured according to a previously described method^39^, the system setup for which is illustrated in Fig. 3. Briefly, we measured broadband signals from the system, and the inertial cavitation dose (ICD) value was calculated as the root-mean-square (RMS) value of the spectrum between 9.5 MHz and 10.5 MHz. The differential ICD (dICD) value was obtained by subtracting the RMS amplitude of the contrast agent from that of water. The results showed that the dICD value increased gradually when AuNDs were exposed to ultrasound and laser radiation simultaneously (Fig. 4a). In contrast, the dICD value decreased gradually during the 5-minute exposure to ultrasound, while the value was difficult to calculate when AuNDs were exposed only to laser radiation. We also exposed AuMBs to ultrasound and laser radiation simultaneously, and found that the dICD value was also gradually reduced during the 5-minute exposure. A stronger and gradually increasing cavitation effect was found for AuNDs relative to AuMBs under exposure to ultrasound and laser radiation simultaneously. These data together indicate that the enhanced cavitation effect is triggered when AuNDs are exposed to ultrasound and laser radiation simultaneously, and it also suggests that the cavitation is predominantly induced by the ultrasound rather than the laser radiation.

Based on the property that the cavitation effect of a contrast agent can be triggered by ultrasound, utilizing contrast agent as the drug carrier has been broadly applied for increasing the rate of drug release in order to the improve the therapeutic efficiency^2^. Our data indicated that the cavitation effect was increased by utilizing AuNDs as the contrast agent followed by exposure to ultrasound and laser radiation simultaneously, and thus we hypothesized that the rate of AuNRs release should also be increased under such treatment. By counting the residual integrated number of AuNDs and measuring the OD_808_ value of released AuNRs for three stimulation methods, we found that exposing AuNDs to ultrasound and laser radiation simultaneously indeed caused the highest destruction ratio and thus enhanced the rate of AuNRs release in comparison with exposure to ultrasound only or laser radiation only (Fig. 4b,c). Furthermore, for exposure to ultrasound and laser radiation simultaneously, the destruction ratio of AuNDs was also significantly higher than that of AuMBs under the same treatment, thus yielding a higher rate of AuNRs release. Since the AuNDs need to be vaporized before being destroyed by ultrasound, it is reasonable that AuMBs showed a higher destruction ratio than AuNDs exposed to ultrasound only, and this also suggested that AuNDs were more stable than AuMBs. However, after AuNDs and AuMBs were exposed to ultrasound, no significant increment in the rate of AuNRs release was found in AuMBs. Nevertheless, the trend was for the rate of AuNRs release to increase in direct proportion to the destruction ratio. These data collectively indicate that the cavitation effect is enhanced only when AuNDs are exposed to ultrasound combined with laser radiation, which thus improves the rate of AuNRs release. To investigate the relationship between the release of AuNRs and the heating efficiency, different concentrations of free AuNRs were treated by laser radiation for 5 minutes, and the final temperatures were measured. The result showed that the elevation of the temperature was highly correlated with the concentration of AuNRs, which the R-squared was 0.99211 of the regression line (Fig. 4d).

### Plasmonic photothermal therapeutic efficiency

**Figure 5:**
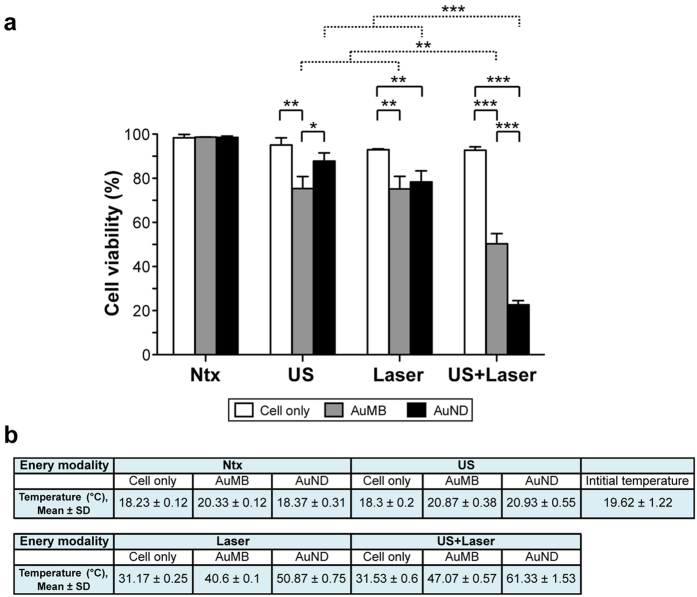
Cell viability measurements. **(a)** Cell viability was determined after contrast-agent-treated cells were exposed to the indicated energy modalities. Statistically significant differences between two independent groups were determined using the statistical methods described in the Methods. Data are mean and SD values. *p < 0.05; **p < 0.01; ***p < 0.001. (**b**) Final temperatures measured after contrast-agent-treated cells were exposed the indicated energy modalities. Temperature data are mean and SD values.

Since we demonstrated that exposing AuNDs to ultrasound combined with laser radiation can improve the rate of AuNRs release, we next hypothesized that carrying and releasing more AuNRs to the target tumor site will enhance the photothermal therapeutic efficiency. To test this hypothesis, *in vitro* cell viability was measured after BNL 1ME A.7R.1 mouse liver cancer cells were incubated with AuNDs and AuMBs followed by the application of three energy modalities, and the thermal effects were examined by using a thermocouple. Cell viability was reduced to 75% or 80% after AuMBs- or AuNDs-treated cells were exposed to ultrasound (Fig. 5a), and the temperatures measured after the treatment were 20.87 °C or 20.93 °C, respectively (Fig. 5b). It suggests that the reduced cell viability is due to ultrasound-induced cavitation. Cell viability was reduced to 75% after AuMBs- or AuNDs-treated cells were exposed to laser radiation (Fig. 5a), and the temperatures measured after the treatment were 40.6 °C or 50.87 °C, respectively (Fig. 5b). It suggests that the reduced cell viability is due to the laser radiation-induced thermal effect. The viability of AuNDs-treated cells was significantly lower when cells were exposed to ultrasound and laser radiation simultaneously compared to either energy modality alone (Fig. 5a). Although the same result was obtained for AuMBs-treated tumor cells, it was clear that it was significantly more feasible to reduce the cell viability of AuNDs-treated tumor cells by exposing them to ultrasound and laser radiation simultaneously. Notably, after cells were exposed to ultrasound and laser radiation simultaneously, the viability of AuNDs-treated cells was significantly reduced from 85% or 50% to 20% in comparison with control cells (no contrast agents treatment) or AuMBs-treated cells. As shown in Fig. 5b, the temperature measured after AuNDs-treated cells were exposed to ultrasound and laser radiation simultaneously was increased to 61.33 °C that was obviously higher than other groups. This represents conclusive evidence that tumor cells are effectively killed by treatment with AuNDs followed by exposure to ultrasound and laser radiation simultaneously *in vitro*, and it suggests that ultrasound-induced cavitation and the laser radiation-induced thermal effect exert the synergistic effect on reducing the cell viability of AuNDs-treated cells.

**Figure 6:**
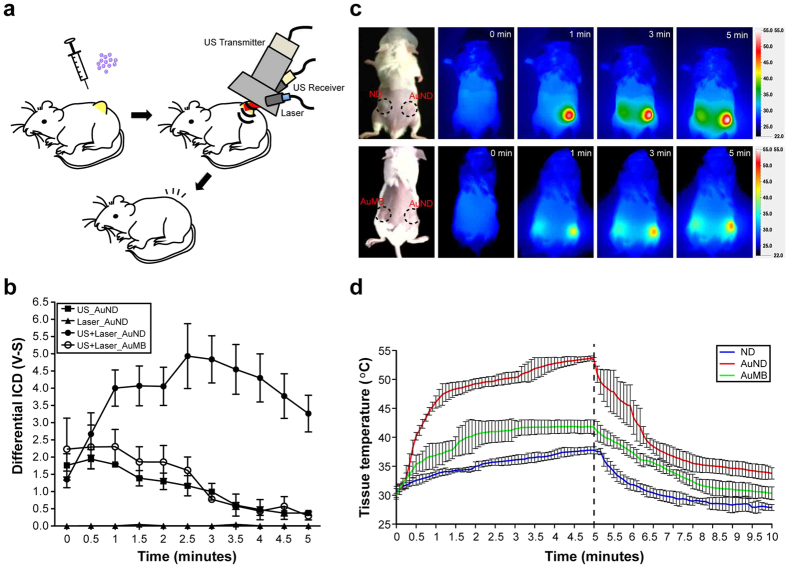
AuNDs-assisted PPTT *in vivo*. (**a**) Schematic of the procedure for applying AuNDs-assisted PPTT to tumor-bearing mice. (**b**) The use of a holding device to ensure that the laser and two ultrasound transducers were co-focused, followed by the same electrical system setup, meant that dICD could be measured *in vivo*. Upon exposure to ultrasound and laser radiation simultaneously for 2.5 minutes, the dICD value was enhanced by 3.1 fold for AuNDs compared to AuMBs with the same treatment, and it was enhanced by 4.2 fold compared to AuNDs exposed to ultrasound only. The enhancement increased to around 10 fold even when the dICD value of the AuNDs with dual energy application has gradually decreased during the last 2.5 minutes. The time zero (t = 0) labeled at the X axis is the starting time to transmit the ultrasound signal from the 1 MHz transmitting transducer, and the amount of cavitation signals are collected by the 10 MHz receiving transducer with a13-seconds delayed. (**c**) Frames from the infrared thermal imaging. (**d**) Real-time temperature recording of the treated tumors during contrast-agent-assisted PPTT. The dotted line indicates the time when exposed to the laser radiation was stopped.

To further understand the therapeutic efficiency *in vivo*, we designed a holding device that allowed an ultrasound transmitter, ultrasound receiver, and laser probe to be co-focused on the targeted tumor site. A schematic of the *in vivo* application method is shown in Fig. 6a. Briefly, after intratumorally injecting AuNRs-encapsulated contrast agent via a needle, the holding device with the instruments set up as described above was placed over the target tumor for applying treatment and for measuring the cavitation signals. The treatment was applied for 5 minutes each time, and this was repeated every 3 days for a total treatment course of 12 days. This setup allowed the cavitation signals to be successfully received from the subcutaneously stimulated implanted liver tumor. The received broadband signals were further analyzed using the same method described in Fig. 4a. Consistent with the *in vitro* results shown in Fig. 4a, the *in vivo* dICD value of AuNDs exposed to ultrasound and laser radiation simultaneously also gradually increased as time passed, while it decreased with time when AuNDs were exposed to ultrasound only or AuMBs were exposed to ultrasound and laser radiation simultaneously. Similarly, the dICD value of laser-exposed AuNDs remained difficult to calculate (Fig. 6b). To examine the thermal effects induced by AuNDs-assisted PPTT, infrared thermal imaging and a thermocouple were applied to measure the temperature of the contrast-agent-treated tumor during the therapy. For infrared thermal imaging, NDs, AuNDs, and AuMBs were individually injected into one side of implanted tumors followed by exposure to ultrasound and laser radiation simultaneously for 5 minutes, during which infrared thermal images were obtained every minute. The results showed that the temperature elevation during the exposure was greatest for AuNDs-treated tumors, with the temperature reaching over 50 °C after 5 minutes of stimulation (Fig. 6c). In contrast, the temperatures measured in NDs- and AuMBs-treated tumors were only elevated to around 35 °C and 42 °C, respectively. Since infrared thermal imaging can only measure the surface temperature, we inserted a thermocouple into the tumor beneath the treatment site to record the intratumor temperature achieved during the treatment. Consistent with the results of infrared thermal imaging, treatment with AuNDs followed by exposure to ultrasound and laser radiation simultaneously could effectively and rapidly elevate the tumor temperature to over 50 °C. Moreover, treatment with either NDs or AuMBs followed by the same excitation method could not elevate the tumor temperature as effectively as when using AuNDs. Together these data indicate that the thermal effect of photothermal therapy was successfully enhanced by applying AuNDs to the tumor followed by exposure to ultrasound and laser radiation simultaneously. Under such treatment, the temperature was sufficiently elevated to cause irreversible thermal damage to the tumor and thus increase the probability of enhancing the photothermal therapeutic efficiency. The enhancement of both the cavitation and thermal effects suggests that enhanced sonoporation is also possible in *in vivo* treatment.

**Figure 7:**
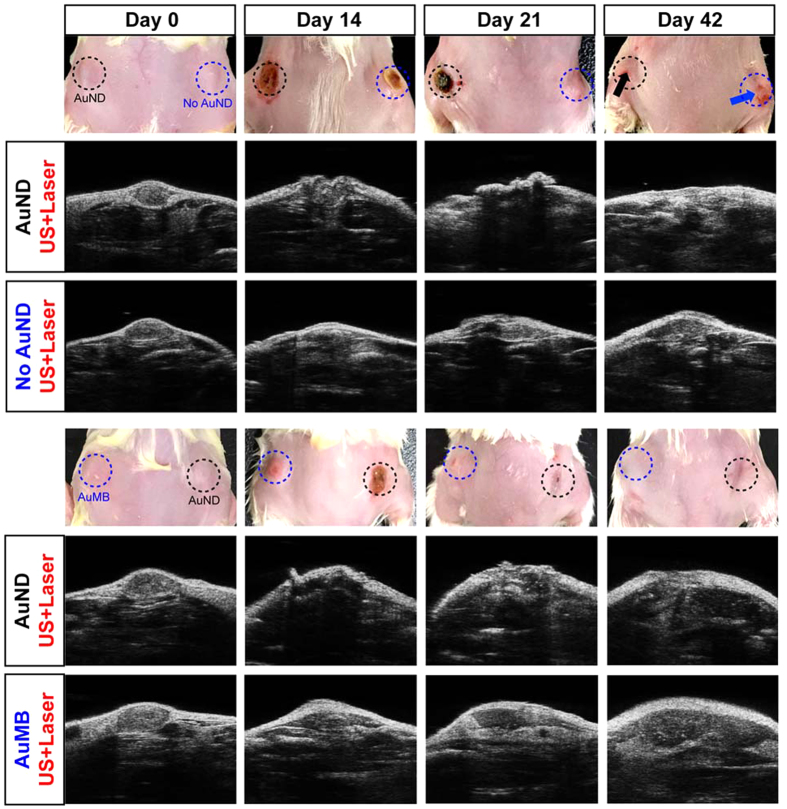
Enhanced therapeutic efficiency in tumors with AuNDs-assisted PPTT. Ultrasound B-mode imaging at the indicated treatment days shows that when tumors exposed to laser radiation and ultrasound simultaneously, the enhanced PPTT efficiency was observed in tumors loaded with AuNDs in compare to tumors loaded with AuMBs or without contrast agent loading.

The implanted tumor was monitored using B-mode imaging every 7 days during PPTT. The images in the top row of Fig. 7 show that AuNDs-treated tumors exhibited a larger scale of injury after PPTT for 14 days compared to the tumors without AuNDs treatment. According to the B-mode image of an AuNDs-treated tumor, such large-scale injury effectively caused the tumor tissue to contract and deform due to thermal denaturation of protein induced by PPTT-induced heating. One week later (on day 21), an obvious scar had formed on the AuNDs-treated tumor, which resulted in a severe imaging artifact appearance during the B-mode imaging. Although a scar that also formed in the tumor without AuNDs treatment generated a small ultrasound imaging artifact, that scar was smaller and it was clearly evident that not all of the tumor tissue had been successfully damaged. After applying PPTT to the implanted tumors for 1.5 months (day 42), the AuNDs-treated tumor was fully cured, whereas the tumor that had not received AuNDs treatment had relapsed and actually enlarged. The similar findings were also found in compared the AuNDs-treated tumor with AuMBs-treated tumor, which the AuNDs-treated tumor was fully cured, whereas the AuMBs-treated one was relapsed and enlarged after applying PPTT. These data demonstrate that treating a tumor with AuNDs followed by PPTT severely damages tumor tissue and thus successfully destroys the tumor, while a tumor that does not receive AuNDs treatment or treats with AuMBs displays comparatively minor damage that cannot destroy it.

### Histological and cellular examination of treated tumor

**Figure 8:**
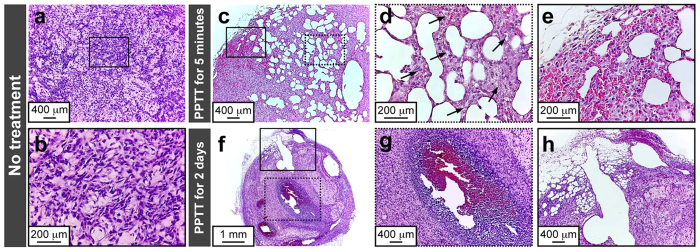
Histological investigation. (**a**) Tumor without any treatment. (**b**) Magnified image of the area indicated by the rectangle in (**a**). (**c,f**) Tumors after being treated with AuNDs-assisted PPTT for either 5 minutes or 2 days. (**d,e,g,h**) Magnified images of the areas indicated by the solid and dotted-dashed rectangles in (**c**,**f**).

To examine the histological damage induced by AuNDs-assisted PPTT on a tumor, tumors treated with AuNDs followed by PPTT for 5 minutes or 2 days and tumors that did not receive AuNDs treatment were dissected out and stained with hematoxylin and eosin (H&E) for histological assessments. Microscopy investigations revealed the creation of several bubble-like structures along with hemorrhage and karyorrhexis (arrows in Fig. 8a–e) in AuNDs-treated tumor tissue after PPTT for 5 minutes. In contrast, tumors that did not receive any treatment did not show any bubble-like structure, hemorrhage, or karyorrhexis. Moreover, after PPTT for 2 days, heat-induced coagulative necrosis that displayed hemorrhage, pyknosis, and karyolysis was found in AuNDs-treated tumor tissue (Fig. 8f). The magnified image in Fig. 8g shows that some hollow spaces were created in the damaged areas, suggesting that coagulative necrotic tumor cell corpses had been cleared. These data show that coagulative necrosis resulting from thermal ablation is the main reason for the death of tumor cells after treatment with AuNDs followed by PPTT.

**Figure 9:**
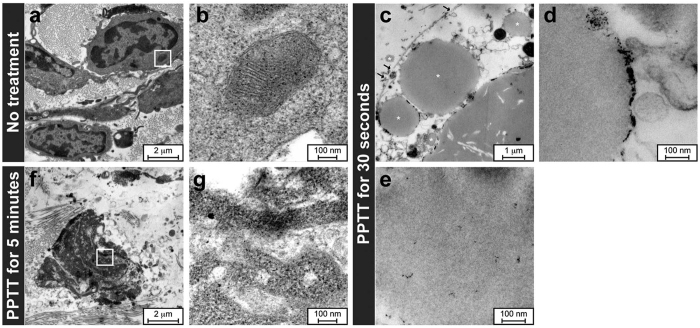
TEM for cellular investigation. (**a**) Cells in a tumor that was not treated. (**b**) Magnified image of the area indicated by the rectangle in (**a**). (**c–e**) AuNDs located in tumors treated with PPTT for only 30 seconds. Black arrows in (**c**): disrupted sites of cellular membrane. White stars in (**c**): intact AuNDs. (**f**) After AuNDs-assisted PPTT is performed for 5 minutes, no droplets or bubbles were found, and the cellular structures no longer appeared integrated. (**g**) Magnified image of the area indicated by the rectangle in (**f**). Many free AuNRs are evident throughout the fractured cells.

TEM was used to evaluate the changes in AuNDs and cellular damage resulting from AuNDs-assisted PPTT in treated tumors. As shown in Fig. 9a, untreated tumors appeared with integrated cellular structures; the magnified organelle shown in Fig. 9b was an integrated mitochondrion located within the square in Fig. 9a. After AuNDs located in tumor were exposed to PPTT for 30 seconds, it was enlarged to several microns in diameter—as expected due to the vaporization—with several cracks appearing on the outer membrane of the droplets, in contrast to AuNDs (labeled by white stars) that had not been expanded to disrupt the membrane integrity (Fig. 9c). Furthermore, sonoporation-induced cellular plasma membrane disruption was observed and pointed out by black arrows in Fig. 9c. Consistent with a previously reported concept^28^, AuNRs were subsequently pushed to the membrane of AuNDs or expelled from AuNDs during vaporization (Fig. 9d). In some nanosized droplets, AuNRs were still encapsulated into the HSA shell due to the received energy being insufficient for vaporization to push the AuNRs out from AuNDs (Fig. 9e). After PPTT was applied for 5 minutes, most of the AuNDs had been destroyed and the cellular structure was disrupted (Fig. 9f), with damaged organelles evident under higher magnification and the AuNRs being dispersed throughout the tumor tissue (Fig. 9g). These results provide direct evidence of changes in AuNDs during PPTT *in vivo* and damage at the cellular level, and they help us to characterize the *in vivo* loaded AuNDs and understand the potential mechanism of AuNDs-assisted PPTT *in vivo*.

## Discussion

Here we have reported on a technology that significantly enhances the efficiency of photothermal therapy via highly potent vaporization-assisted sonoporation. By combining optical droplet vaporization and acoustic cavitation of AuNDs, we successfully achieved synergistic physical effects that enhance the extravasation rate of photoreactive AuNRs in a tumor through sonoporation. This process allows more photoreactive AuNRs to be delivered to the tumor so as to enhance the degree of photothermal hyperthermia. This methodology involves applying enhanced sonoporation combined with the subsequent thermal effect, and we have successfully demonstrated its value both in terms of *in vitro* tumor cell viability and *in vivo* tumor treatment efficiency.

The droplet-to-bubble phase transition of NDs (vaporization) is believed to be the main physical mechanism underlying the subsequent induction of cavitation. In addition, cavitation from the destruction of MBs further induces the AuNRs released from AuNDs. The B-mode images shown in Fig. 2 indicate that the AuNDs were vaporized when they received energy from the laser only or the laser combined with ultrasound. However, in comparison to AuNDs receiving energy from ultrasound only, no obvious vaporization was triggered. It is reasonable to assume that the triggered vaporization in our case was due to optical droplet vaporization. Since the NDs include low-boiling-point DDFC liquid and the AuNRs can absorb optical energy, vaporization is easily triggered via heating. Several studies have shown that the vaporization threshold of acoustic droplets can be affected by changing various acoustic parameters including the pulse duration, number of pulse cycles, sonication frequency, and acoustic pressure. The materials used to construct the shell and core of the droplet, the droplet size, and the loading concentration of the droplets were also taken into account^20^,^40^,^41^,^42^,^43^,^44^,^45^. It has been shown that when smaller droplets are formed, larger negative acoustic pressures are required for vaporization^40^,^41^,^42^,^43^. For example, the acoustic vaporization thresholds of 7.2-μm and 2-μm droplets sonicated at 2.855 MHz were reportedly 2 MPa and 4 MPa, respectively^40^. In addition, inertial cavitation is detected before the vaporization of smaller droplets due to the vaporization threshold being higher than the inertial cavitation threshold. Because the droplets used in this study were only around 300 nm in diameter and the peak negative pressure was only 526 kPa, these studies support our finding of no obvious vaporization of AuNDs and that the inertial cavitation was still observed during stimulation by acoustic energy only. Since the vaporization of AuNDs decreases the inertial cavitation threshold, the images shown in the first row of Fig. 2 had lower brightness due to the inertial cavitation occurs once AuNDs are vaporized to AuMBs. Our findings agree with several recent studies that report the cavitation threshold of a gold-coated perfluorohexane nanoemulsion is reduced when ultrasound and laser radiation are applied simultaneously in comparison with exposure to ultrasound or laser radiation alone^46^,^47^,^48^. The mechanism they used is applying the temporal control of the laser stimulation at the peak negative pressure of ultrasound to reduce the cavitation threshold. They also trigger both nanoemulsion vaporization and inertial cavitation via combining pulsed laser and ultrasound to disrupt the blood clot without heating. Although we both apply the laser and ultrasound on therapy, since the intention of our study is to perform the photothermal therapy, the use of CW laser was implemented. In the present study, although the optically triggered vaporization of laser-exposed AuNDs was obviously observed, we found it difficult to detect the cavitation effect both *in vitro* and *in vivo*. According to the results shown in the Figs 2,4a and 6b, it suggests the main role of the CW laser we used is to trigger the vaporization and deliver the thermal effect not cavitation, unlike the role of the pulsed laser in other studies being to trigger both vaporization and cavitation at the peak negative acoustic pressure. Moreover, the cavitation amount was markedly increased when ultrasound and laser radiation were applied to AuNDs simultaneously. We thus conclude that the main energy source for triggering the vaporization of AuNDs is laser radiation, with ultrasound playing a crucial role in inducing inertial cavitation.

More importantly, comparison of the AuNDs destruction ratio and AuNRs releasing rate induced by AuMBs with ultrasound application only and by AuNDs exposed to ultrasound and laser radiation simultaneously reveals that both the destruction ratio and the OD values of the AuNRs released from AuNDs exposed to two types of energy were more than twofold higher than those for the AuMBs exposed only to ultrasound. This demonstrates that the AuNDs destruction ratio and AuNRs releasing rate is substantially increased through such AuNDs-assisted photoacoustic stimulation. These findings strongly support that synergistic AuNDs destruction and AuNRs release is attributable to AuNDs being stimulated by two energy sources to trigger optical vaporization and acoustic cavitation simultaneously. Furthermore, the AuNRs employed here not only to absorb optical energy for the triggering of vaporization, but also the therapeutic agents of PPTT. Such a self-activating property of AuNRs combined with synergistic cavitation and thermal effect thus significantly improved the therapeutic efficiency. More contribution to improve the AuNDs destruction and the subsequent AuNRs release underlies further investigation of the stability of the droplets depends on several other parameters.

The histological investigations revealed clear damage once the AuNDs-treated tumor tissue had been stimulated by both acoustic and optical energy modalities. One of the cellular phenotypes, karyorrhexis, was found in the tissue sections, which indicates these dying cells showed fragmented nuclei and a damaged cellular structure. Since karyorrhexis is one of the characteristics of apoptosis, this result suggests that PPTT induces both hyperthermia-induced coagulative necrosis and apoptosis to kill the tumor. Some dilated cave structures were also observed within the treated tumor tissue, which could have been created by the mechanical forces associated with the continuous vaporization-assisted sonoporation. We also found acute hemorrhage occurs due to the thermal-induced coagulative necrosis, which blood escapes from the thermal destroyed blood vessel, and few days later, macrophages, neutrophils and lymphocytes would gather surrounding the thermal coagulative lesion due to the inflammatory reaction^49^. The hemorrhage may also occur due to the inertial cavitation has also been observed^50^. Some fragmented cell pieces were found surrounding the cave structures that were consistent with the fractured cellular phenotype found in the TEM images. These dying cells also hint that mechanical forces such as the shock pressures induced when bubbles collapse violently were the reason for damage to part of the tumor cells and also the *in vivo* results of the vaporization-assisted sonoporation. Based on the TEM findings, we can draw some further conclusions. First, fully vaporized AuNDs had disappeared after the tumor had received the 5 minutes of PPTT, suggesting that they had collapsed due to cavitation or the dissolution due to the gas diffusion^51^. Second, the photoreactive AuNRs expelled from collapsed AuNDs were found everywhere surrounding the cellular fragments, suggesting that substantial numbers of free AuNRs are indeed retained within the tumor tissue for heating. Third, consistent with the H&E staining results, severe irreversible cellular damage was also evident in the TEM images. All of these conclusions support the successfully induction of photothermal therapy *in vivo*. According to a review paper in discussing the cytotoxicity of gold nanoparticles, the toxic dose is 4.26 mg gold/kg body weight in BALB/c mice^52^. Thus, the total amount of AuNRs we injected during the whole treatment course was calculated by using the regression line in Fig. 4d. Since the total amount of AuNRs we injected was only 1.36 nM (1.2 ng) per mouse (body weight is around 25 g), we deduced that the dose we used is non-toxic in mice.

In accordance with the findings of previous studies, cavitation that occurs around heated nanoparticles induces cell death in the area where the nanoparticles are located due to disruption by strong mechanical forces^53^,^54^. This is an important reason supporting the application of AuNDs-assisted photoacoustic stimulation to enhance the efficiency of sonoporation-directed tumor therapy. The utilization of specific antibody-conjugated contrast agents for targeted tumor therapy has been studied for a long time, since this helps the contrast agent to specifically target and concentrate the stimulation on the targeted tumor for locally releasing the carried drug or metallic nanoparticles from acoustically destroyed contrast agent^15^,^32^,^55^. Accordingly, to further improve AuNDs-assisted photothermal tumor therapy, applying a targeted-AuNDs approach followed by intravenous injection can enhance the sonoporation of AuNDs and the subsequent EPR effect at the target site.

Moreover, we used the co-focused method to successfully analyze the cavitation effect *in vivo* for the first time. The results support the further assessment of *in vivo* sonoporation efficiency with different energy sources applied to the AuNDs. The markedly enhanced cavitation effect measured when using the co-focused method combined with rapid thermal increase detected in the AuNDs-treated tumor allowed us to conclude that the enhanced efficiency of PPTT is due to the high potency of vaporization-assisted sonoporation. The use of the co-focused method can help to further understand the physical parameters and the biological effects of AuNDs-assisted photoacoustic stimulation. Thus, the delivery and accumulation of AuNPs in targeted tumor lesions via the synergy of diverse mechanisms could be further optimized.

**Figure 10:**
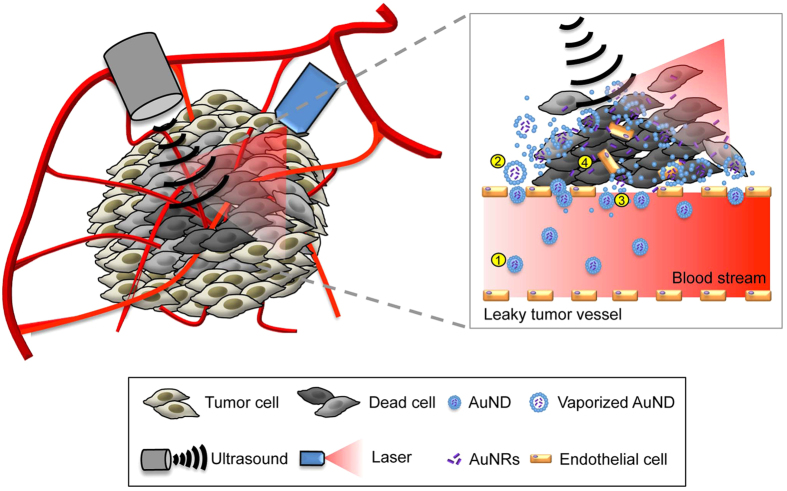
AuNDs-assisted PPTT. Schematic of the concept and mechanism of AuNDs-assisted PPTT for a tumor. A detailed description is presented in the last paragraph of the Results. Briefly, in the first step, after AuNDs are loaded into the tumor, the EPR effect of the AuNDs in the tumor is increased due to their smallness. In the second step, optically triggered optical droplet vaporization causes expansion of AuNDs and also instability of their HSA shell. In the third step, acoustic cavitation is induced and causes the violent collapse of the vaporized AuNDs, and the subsequent more-permeable defects allow more AuNDs to be delivered to the tumor tissue. In the fourth step, the enhanced EPR effect of AuNDs promotes more AuNRs to be expelled from AuNDs and subsequently delivered to the tumor, thereby improving the thermal ablation efficiency of PPTT.

The potential mechanism is illustrated schematically in Fig. 10. In the first step, when AuNDs are loaded into a tumor and flow into a leaky vessel located inside the tumor, the nanosized AuNDs can easily pass through the endothelial gaps (with a range between 0.2 to 1.2 μm in mouse tumors) to improve the EPR effect of AuNDs. Since the AuNDs are relative stable carriers, this enhances the delivery of AuNRs to tumor tissue. In the second step, laser-induced optical droplet vaporization is induced to expand the AuNDs to several microns in diameter due to the absorbance of the optical energy by the encapsulated AuNRs. Meanwhile, the surface of the HSA shell of the expanded AuNDs becomes unstable due to the cracks, resulting in leakage of the AuNRs and facilitating the activation of the subsequent acoustic cavitation. In the third step, AuNDs were fully vaporized to AuMBs. Since the membrane of the HSA shell becomes even more unstable, acoustic cavitation is easy to trigger to induce the collapse of AuMBs. AuNRs are subsequently released from the violently collapsed AuMBs to expand the PPTT-affected area. The shock wave generated by the violently collapsed AuMBs also induces more-permeable defects in the endothelial walls that allow more AuNDs to pass through during the next cycle of energy application. In the final step, by continuously applying the AuNDs-assisted PPTT for a while, tumor tissue is thermal ablated by optically induced hyperthermia via heating of the AuNRs released from AuNDs. AuNRs play a self-activating role in this process by not only enhancing the vaporization in the first three steps but also for inducing thermal ablation in the last step.

Several applications involving the use of NDs for imaging contrast enhancement, drug delivery, and gene introduction have been reported previously, but no previous study has demonstrated NDs-assisted PPTT based on the significant enhancement of sonoporation. This study is the first to successfully utilize triggered AuNDs vaporization to enhance sonoporation both *in vitro* and *in vivo* so as to achieve highly effective delivery of photothermal treatment. Furthermore, we also investigated various possible mechanisms for the effects of ultrasound and laser radiation on AuNDs-treated tumors during sonoporation-assisted treatment delivery. The method can also be applied to other areas such as sonoporation-assisted gene therapy.

## Methods

### Synthesis of contrast agents

AuNDs were synthesized using an emulsion method, and AuMBs were synthesized as described previously^7^. The synthesis of AuNDs involved premixing AuNRs with an absorption peak at 808 nm and ice-cold liquid C_5_F_12_ with a low boiling point of 29 °C in 20% HSA (Octapharma, Switzerland) in a glass bottle, and then exposing the mixture to ultrasound by a digital sonicator with a cup-horn sonotrode (Branson, USA). The solution was exposed to three cycles of 5-minute sonication at room temperature followed by 5 minutes of rest on ice. After centrifugation at 1800 rpm for 3 minutes twice, AuNDs with diameters of 300 ~ 500 nm were isolated. The size of the AuNDs was accurately measured using two devices: a Coulter MultiSizer III (Beckman Coulter, USA) and a Zetasizer Nano ZS (Malvern Instruments, UK).

### B-mode imaging

B-mode imaging was used to investigate AuNDs vaporization by replacing the receive transducer with a commercial transducer (10L and LOGIQ 500, GE Medical Systems, USA) in the system setup illustrated in the Fig. 3. For tracing the *in vivo* tumor therapeutic efficiency in tumor-bearing mice, a high-frequency ultrasound system (Prospect, S-Sharp, Taiwan) was used for B-mode imaging of treated tumors. Before performing B-mode scanning, the hair distributed around the tumor was gently removed using hair-remover cream, and the mice were continuously anesthetized by 1.5–2.0% isoflurane inhalant mixed with 1-l/min 100% oxygen during the scanning procedure.

### ICD value measurements and system setup

The system setup in this study is depicted in Fig. 3. For *in vitro* ICD value measurements, a 2% agar phantom with loaded contrast agent was used. A continuous-wave laser (808 nm; Newport, USA) was applied for optical stimulation at a power of 2.6 W (intensity: 2 W/cm^2^) as measured by a power meter. A 1-MHz ultrasound transducer (25.4 mm in diameter and with a focal length of 49.8 mm; V302, Panametrics-NDT, USA) was applied for acoustic stimulation, and a 10-MHz transducer (V312, Panametrics-NDT) was used to detect the cavitation signals. For *in vivo* ICD value measurements, a laser and two ultrasound transducers were placed in a plastic holder such that they were co-focused during the application. The transmit waveform was produced by a digital-to-analog converter (CompuGen 1100, GaGe Applied Technologies, Canada) in the computer to allow adjustment of the frequency, number of cycles, and pulse repetition frequency (PRF). The emitted signals were amplified by an RF power amplifier (250A250A, Amplifier Research, USA), and the received signals were fed to a preamplifier (Pulser/Receiver 5072PR, Panametrics-NDT) and processed by an analog-to-digital converter (CompuScope 12100, GaGe Applied Technologies) in the computer. MATLAB software (Mathworks, USA) was used for analyzing the differential ICD value. The acoustic pressure of the transmitting 1-MHz ultrasound transducer was calibrated using a needle-type hydrophone (MHA9-150, FORCE Technology, Denmark). Cavitation was induced in this study under a fixed peak rarefaction acoustic pressure of 526 kPa, and allowing for the tissue attenuation, the maximum peak rarefaction acoustic pressure would have been around 425 kPa (mechanical index = 0.425). The other fixed parameters used in this study were as follows: 6 × 10^9^ loaded AuNDs, pulse duration of 10 cycles, 150 pulses, and PRF of 100 Hz.

### Destruction ratio and efficiency of AuNRs release

The measure the AuNDs or AuMBs destruction ratio and the efficiency of AuNRs release, 6 × 10^9^ of contrast agents were loaded into a 2-ml Eppendorf tube and placed in a 2% agar phantom. The destruction ratio was calculated by comparing the numbers of AuNDs or AuMBs between before and after exposure to laser radiation only, ultrasound only, or ultrasound and laser radiation simultaneously as measured using a Coulter MultiSizer III device. To measure the efficiency of AuNRs release, AuNRs released from AuMBs or AuNDs were filtered through glass-fiber filter paper with 300-nm pores (GF-75, Advantec, Japan), and then the OD value of the free AuNRs was assessed using a plate reader (Enspire 2300, PerkinElmer, USA) after subtracted the background from the filtrate of non-treated AuNDs. The initial OD values of non-treated AuMBs and AuNDs were assessed after subtracted the background from the same numbers of MBs or NDs.

### Cell culture and cell viability assay

BNL 1ME A.7R.1 mouse liver cancer cells were cultured in DMEM containing 10% fetal bovine serum and a 1% penicillin-streptomycin solution at 37 °C in a 5% CO_2_ incubator. To measure the *in vitro* cellular photothermal therapeutic efficiency, 10^6^ of cells were trypsinized off the culture dishes and mixed well with 6 × 10^9^ of AuNDs or AuMBs. The mixtures were then loaded into the agar phantom followed by immediately exposure to laser radiation only, ultrasound only, or ultrasound and laser simultaneously for 5 minutes. After applying the energy stimulation, cells were removed from the phantom and staining with trypan blue. Stained cells were mixed well and evenly spread on a hemacytometer to measure cell viability with the aid of a microscope.

### Animal model, temperature recording, and *in vivo* PPTT

The mouse tumor model was constructed by resuspending a cell pellet containing 5 × 10^6^ BNL 1ME A.7R.1 liver cancer cells with 100 μl of Matrigel and then injecting the mixture subcutaneously into one side of the back of an anesthetized 6-week-old male BALB/c mouse. After injecting the tumor cells, the mice were immediately moved into a warm environment to avoid temperature loss during anesthesia. After implantation for 2 weeks the tumors had grown to 3 mm in diameter and were used for AuNDs-assisted PPTT. NDs, AuNDs, or AuMBs (6 × 10^9^) were then loaded intratumorally into the mice followed by the application of energy stimulation. Before applying PPTT, the hair distributed around the loading site was gently removed using hair-remover cream, and the mice were continuously anesthetized by 1.5–2.0% isoflurane inhalant mixed with 1-l/min 100% oxygen throughout the application period. A needle-type thermocouple (Thermocouple Probe Model HYP1, OMEGA, USA) was used to record the temperature of treated tumors during PPTT, and thermal imaging was performed using a portable infrared thermal imaging camera (Thermo Gear G100EX, Avio NEC, Japan). All animals were maintained in accordance with the regulations set by the Medical School of National Taiwan University, and all animal experiments were conducting followed the institutional guidelines and under a protocol approved by the Institutional Animal Care and Use Committee.

### TEM and H&E histological investigations

Samples were investigated using TEM and cTEM at the electron microscopy facility of Academia Sinica. For H&E histological investigations, samples were fixed in 4% paraformaldehyde followed by processing with dehydration and subsequent embedding in paraffin. Paraffin-embedded samples were sectioned at a thickness of 5 μm, and the sections were then processed by deparaffinization and rehydration, stained with H&E, and investigated using microscopy.

### Statistical analyses

All statistical data were obtained from three separate experiments. For *in vivo* AuNDs-assisted PPTT and ICD value measurements, seven sides with implanted tumors were used for data collection and further analysis. Statistical data were calculated as mean ± SD values, and statistical significance between two experimental groups was analyzed using the two-tailed unpaired Student’s *t* test.

## Additional Information

**How to cite this article**: Liu, W.-W. *et al.* Nanodroplet-Vaporization-Assisted Sonoporation for Highly Effective Delivery of Photothermal Treatment. *Sci. Rep.* **6**, 24753; doi: 10.1038/srep24753 (2016).

## References

[b1] Aryal M., Arvanitis C. D., Alexander P. M. & McDannold N. Ultrasound-mediated blood-brain barrier disruption for targeted drug delivery in the central nervous system. Adv. Drug Deliv. Rev. 72, 94–109 (2014). 24462453 10.1016/j.addr.2014.01.008 PMC4041837

[b2] Lammertink B. H. *et al.* Sonochemotherapy: from bench to bedside. Front. Pharmacol. 6, 138 (2015). 26217226 10.3389/fphar.2015.00138 PMC4498442

[b3] VanBavel E. Effects of shear stress on endothelial cells: possible relevance for ultrasound applications. Prog. Biophys. Mol. Biol. 93, 374–383 (2007). 16970981 10.1016/j.pbiomolbio.2006.07.017

[b4] Wu J. Theoretical study on shear stress generated by microstreaming surrounding contrast agents attached to living cells. Ultrasound Med. Biol. 28, 125–129 (2002). 11879959 10.1016/s0301-5629(01)00497-5

[b5] Fan Z., Kumon R. E. & Deng C. X. Mechanisms of microbubble-facilitated sonoporation for drug and gene delivery. Ther. Deliv. 5, 467–486 (2014). 24856171 10.4155/tde.14.10 PMC4116608

[b6] Delalande A., Kotopoulis S., Rovers T., Pichon C. & Postema M. Sonoporation at a low mechanical indiex. Bubble Sci. Engin. Technol. 3, 3–12 (2011).

[b7] Prentice P., Cuschieri A., Dholakia K., Prausnitz M. & Campbell P. Membrane disruption by optically controlled microbubble cavitation. Nature Phys. 1, 107–110 (2005).

[b8] Postema M., Kotopoulis S., Delalande A. & Gilja O. H. Sonoporation: Why microbubbles create pores. Ultraschall Med. 33, 97–98 (2012).

[b9] Delalande A., Kotopoulis S., Postema M., Midoux P. & Pichon C. Sonoporation: mechanistic insights and ongoing challenges for gene transfer. Gene 525, 191–199 (2013). 23566843 10.1016/j.gene.2013.03.095

[b10] Ferrara K., Pollard R. & Borden M. Ultrasound microbubble contrast agents: fundamentals and application to gene and drug delivery. Annu. Rev. Biomed. Eng. 9, 415–447 (2007). 17651012 10.1146/annurev.bioeng.8.061505.095852

[b11] Tinkov S., Bekeredjian R., Winter G. & Coester C. Microbubbles as ultrasound triggered drug carriers. J. Pharm. Sci. 98, 1935–1961 (2009). 18979536 10.1002/jps.21571

[b12] Wang Y. H., Liao A. H., Chen J. H., Wang C. R. & Li P. C. Photoacoustic/ultrasound dual-modality contrast agent and its application to thermotherapy. J. Biomed. Opt. 17, 045001 (2012). 22559675 10.1117/1.JBO.17.4.045001

[b13] Fan Z., Kumon R. E. & Deng C. X. Mechanisms of microbubble-facilitated sonoporation for drug and gene delivery. Ther. Deliv. 5, 467–486 (2014). 24856171 10.4155/tde.14.10 PMC4116608

[b14] Rychak J. J. & Klibanov A. L. Nucleic acid delivery with microbubbles and ultrasound. Adv. Drug Deliv. Rev. 72, 82–93 (2014). 24486388 10.1016/j.addr.2014.01.009 PMC4204336

[b15] Wang Y. H. *et al.* Synergistic delivery of gold nanorods using multifunctional microbubbles for enhanced plasmonic photothermal therapy. Sci. Rep. 4, 5685 (2014). 25023090 10.1038/srep05685 PMC4097346

[b16] Rapoport N. Phase-shift, stimuli-responsive perfluorocarbon nanodroplets for drug delivery to cancer. Wiley Interdiscip. Rev. Nanomed. Nanobiotechnol. 4, 492–510 (2012). 22730185 10.1002/wnan.1176 PMC3482424

[b17] Anderson C. R. *et al.* Ultrasound molecular imaging of tumor angiogenesis with an integrin targeted microbubble contrast agent. Invest. Radiol. 46, 215–224 (2011). 21343825 10.1097/RLI.0b013e3182034fed PMC3075480

[b18] Matsunaga T. O. *et al.* Phase-change nanoparticles using highly volatile perfluorocarbons: toward a platform for extravascular ultrasound imaging. Theranostics 2, 1185–1198 (2012). 23382775 10.7150/thno.4846 PMC3563153

[b19] Sheeran P. S., Luois S., Dayton P. A. & Matsunaga T. O. Formulation and acoustic studies of a new phase-shift agent for diagnostic and therapeutic ultrasound. Langmuir 27, 10412–10420 (2011). 21744860 10.1021/la2013705 PMC3164903

[b20] Kripfgans O. D., Fowlkes J. B., Miller D. L., Eldevik O. P. & Carson P. L. Acoustic droplet vaporization for therapeutic and diagnostic applications. Ultrasound Med. Biol. 26, 1177–1189 (2000). 11053753 10.1016/s0301-5629(00)00262-3

[b21] Strohm E., Rui M., Gorelikov I., Matsuura N. & Kolios M. Vaporization of perfluorocarbon droplets using optical irradiation. Biomed. Opt. Express 2, 1432–1442 (2011). 21698007 10.1364/BOE.2.001432 PMC3114212

[b22] Dayton P. A. & Matsunaga T. O. Ultrasound-mediated therapies using oil and perfluorocarbon-filled nanodroplets. Drug Dev. Res. 67, 42–46 (2006).

[b23] Kripfgans O. D. *et al.* Acoustic droplet vaporization for temporal and spatial control of tissue occlusion: a kidney study. IEEE Trans. Ultrason. Ferroelectr. Freq. Control 52, 1101–1110 (2005). 16212249 10.1109/tuffc.2005.1503996

[b24] Chen C. C. *et al.* Targeted drug delivery with focused ultrasound-induced blood-brain barrier opening using acoustically-activated nanodroplets. J. Control Release 172, 795–804 (2013). 24096019 10.1016/j.jconrel.2013.09.025 PMC3866692

[b25] Moyer L. C. *et al.* High-intensity focused ultrasound ablation enhancement *in vivo* via phase-shift nanodroplets compared to microbubbles. J. Ther. Ultrasound 3, 7 (2015). 26045964 10.1186/s40349-015-0029-4 PMC4455327

[b26] Pajek D., Burgess A., Huang Y. & Hynynen K. High-intensity focused ultrasound sonothrombolysis: the use of perfluorocarbon droplets to achieve clot lysis at reduced acoustic power. Ultrasound Med. Biol. 40, 2151–2161 (2014). 25023095 10.1016/j.ultrasmedbio.2014.03.026 PMC4130783

[b27] Zhu M. *et al.* Treatment of murine tumors using acoustic droplet vaporization-enhanced high intensity focused ultrasound. Phys. Med. Biol. 58, 6179–6191 (2013). 23948709 10.1088/0031-9155/58/17/6179

[b28] Phillips L. C. *et al.* Phase-shift perfluorocarbon agents enhance high intensity focused ultrasound thermal delivery with reduced near-field heating. J. Acoust. Soc. Am. 134, 1473–1482 (2013). 23927187 10.1121/1.4812866 PMC3745500

[b29] Zhou Y. *et al.* Microbubbles from gas-generating perfluorohexane nanoemulsions for targeted temperature-sensitive ultrasonography and synergistic HIFU ablation of tumors. Adv. Mater. 25, 4123–4130 (2013). 23788403 10.1002/adma.201301655

[b30] Zhang M. *et al.* Acoustic droplet vaporization for enhancement of thermal ablation by high intensity focused ultrasound. Acad. Radiol. 18, 1123–1132 (2011). 21703883 10.1016/j.acra.2011.04.012 PMC3152672

[b31] Zhang P. & Porter T. An *in vitro* study of a phase-shift nanoemulsion: a potential nucleation agent for bubble-enhanced HIFU tumor ablation. Ultrasound Med. Biol. 36, 1856–1866 (2010). 20888685 10.1016/j.ultrasmedbio.2010.07.001

[b32] Korpanty G., Grayburn P. A., Shohet R. V. & Brekken R. A. Targeting vascular endothelium with avidin microbubbles. Ultrasound Med. Biol. 31, 1279–1283 (2005). 16176794 10.1016/j.ultrasmedbio.2005.06.001

[b33] Gao D. *et al.* Ultrasound-triggered phase-transition cationic nanodroplets for enhanced gene delivery. ACS Appl. Mater. Interfaces 7, 13524–13537 (2015). 26016606 10.1021/acsami.5b02832

[b34] Burgess M. T. & Porter T. M. Acoustic cavitation-mediated delivery of small interfering ribonucleic acids with phase-shift nano-emulsions. Ultrasound Med. Biol. 41, 2191–2201 (2015). 25979417 10.1016/j.ultrasmedbio.2015.04.002 PMC4466208

[b35] Paproski R. J., Forbrich A., Hitt M. & Zemp R. RNA biomarker release with ultrasound and phase-change nanodroplets. Ultrasound Med. Biol. 40, 1847–1856 (2014). 24792584 10.1016/j.ultrasmedbio.2014.01.011

[b36] Wilson K., Homan K. & Emelianov S. Biomedical photoacoustics beyond thermal expansion using triggered nanodroplet vaporization for contrast-enhanced imaging. Nat. Commun. 3, 618 (2012). 22233628 10.1038/ncomms1627

[b37] Huang X. & El-Sayed M. A. Plasmonic photo-thermal therapy (PPTT). Alexandria J. Med. 47, 1–9 (2011).

[b38] Dickerson E. B. *et al.* Gold nanorod assisted near-infrared plasmonic photothermal therapy (PPTT) of squamous cell carcinoma in mice. Cancer Lett. 269, 57–66 (2008). 18541363 10.1016/j.canlet.2008.04.026 PMC3413727

[b39] Lai C. Y., Wu C. H., Chen C. C. & Li P. C. Quantitative relations of acoustic inertial cavitation with sonoporation and cell viability. Ultrasound Med. Biol. 32, 1931–1941 (2006). 17169705 10.1016/j.ultrasmedbio.2006.06.020

[b40] Schad K. C. & Hynynen K. *In vitro* characterization of perfluorocarbon droplets for focused ultrasound therapy. Phys. Med. Biol. 55, 4933–4947 (2010). 20693614 10.1088/0031-9155/55/17/004

[b41] Kripfgans O. D., Fabiilli M. L., Carson P. L. & Fowlkes J. B. On the acoustic vaporization of micrometer-sized droplets. J. Acoust. Soc. Am. 116, 272–281 (2004). 15295987 10.1121/1.1755236

[b42] Sheeran P. S. *et al.* Decafluorobutane as a phase-change contrast agent for low-energy extravascular ultrasonic imaging. Ultrasound Med. Biol. 37, 1518–1530 (2011). 21775049 10.1016/j.ultrasmedbio.2011.05.021 PMC4450864

[b43] Matsunaga T. O. *et al.* Phase-change nanoparticles using highly volatile perfluorocarbons: toward a platform for extravascular ultrasound imaging. Theranostics 2, 1185–1198 (2012). 23382775 10.7150/thno.4846 PMC3563153

[b44] Lo A. H., Kripfgans O. D., Carson P. L., Rothman E. D. & Fowlkes J. B. Acoustic droplet vaporization threshold: effects of pulse duration and contrast agent. IEEE Trans. Ultrason. Ferroelectr. Freq. Control 54, 933–946 (2007). 17523558 10.1109/tuffc.2007.339

[b45] Kawabata K.-I., Sugita N., Yoshikawa H., Azuma T. & Umemura S.-I. Nanoparticles with multiple perfluorocarbons for controllable ultrasonically induced phase shifting. Japan J. Appl. Phys. 44, 4548–4552 (2005).

[b46] Arnal B. *et al.* Sono-photoacoustic imaging of gold nanoemulsions: Part I. Exposure thresholds. Photoacoustics 3, 3–10 (2015). 25893169 10.1016/j.pacs.2014.12.001 PMC4398805

[b47] Arnal B. *et al.* Sono-photoacoustic imaging of gold nanoemulsions: Part II. Real time imaging. Photoacoustics 3, 11–19 (2015). 25893170 10.1016/j.pacs.2015.01.001 PMC4398795

[b48] Wei C. W. *et al.* Laser-induced cavitation in nanoemulsion with gold nanospheres for blood clot disruption: *in vitro* results. Opt. Lett. 39, 2599–2602 (2014). 24784055 10.1364/OL.39.002599 PMC9008802

[b49] Thomsen S. & Pearce J. A. Thermal damage and rate processes in biologic tissues In Optical-thermal response of laser-irradiated tissue 2nd edn (eds Welch A. J. & van Gemert M. J. C.) Ch. 13, 487–549 (Springer, 2011).

[b50] Xu Z. *et al.* Intracranial inertial cavitation threshold and thermal ablation lesion creation using MRI-guided 220-kHz focused ultrasound surgery: preclinical investigation. J. Neurosurg. 122, 152–161 (2015). 25380106 10.3171/2014.9.JNS14541

[b51] Reznik N. *et al.* The efficiency and stability of bubble formation by acoustic vaporization of submicron perfluorocarbon droplets. Ultrasonics 53, 1368–1376 (2013). 23652262 10.1016/j.ultras.2013.04.005

[b52] Alkilany A. M. & Murphy C. J. Toxicity and cellular uptake of gold nanoparticles: what we have learned so far? J. Nanopart. Res. 12, 2313–2333 (2010). 21170131 10.1007/s11051-010-9911-8 PMC2988217

[b53] Lukianova-Hleb E. Y., Koneva II, Oginsky A. O., La Francesca S. & Lapotko D. O. Selective and self-guided micro-ablation of tissue with plasmonic nanobubbles. J. Surg. Res. 166, e3–e13 (2011). 21176913 10.1016/j.jss.2010.10.039 PMC3042052

[b54] Peeters S. *et al.* Mechanisms of nanoparticle-mediated photomechanical cell damage. Biomed. Opt. Express 3, 435–446 (2012). 22435092 10.1364/BOE.3.000435 PMC3296532

[b55] Morawski A. M., Lanza G. A. & Wickline S. A. Targeted contrast agents for magnetic resonance imaging and ultrasound. Curr. Opin. Biotechnol. 16, 89–92 (2005). 15722020 10.1016/j.copbio.2004.11.001

